# Mental health in children with and without ADHD: the role of physical activity and parental nativity

**DOI:** 10.1186/s13034-025-00859-8

**Published:** 2025-01-18

**Authors:** David Adzrago, Saanie Sulley, Faustine Williams

**Affiliations:** 1 Division of Intramural Research, National Institute on Minority Health and Health Disparities, National Institutes of Health, Bethesda, MD USA; 2 National Healthy Start Association, Washington, DC USA

**Keywords:** Children’s mental health, Immigrant health paradox, Exercise, Immigration, Neurodevelopmental disorder

## Abstract

**Background:**

Physical activity (PA) can improve mental health, including anxiety/depression, in individuals with attention-deficit/hyperactivity disorder (ADHD) with minimal side effects, unlike pharmacotherapy that can result in significant side effects. However, the influence of PA on mental health among children with ADHD is understudied. Also, immigrants tend to have better mental health, but the influence of parental nativity on children’s mental health is unknown. We examined the relationship between PA, parental nativity, and current anxiety/depression among U.S. children with and without ADHD. We also analyzed whether ADHD diagnosis status moderates the relationship between anxiety/depression and PA or parental nativity.

**Methods:**

We used national annual cross-sectional data from the 2016 to 2021 National Survey of Children’s Health to conduct weighted multivariable logistic regression and moderation analyses, with current anxiety/depression status as the outcome variable. The sampling involves selecting households with children and rostering children in the household from each state and the District of Columbia. A parent or caregiver of the selected child completes the surveys. We restricted the analysis to children aged 6–17 years (*N* = 140,977).

**Results:**

The prevalence of current anxiety/depression was higher in children with ADHD diagnosis (37.34%) than those without ADHD diagnosis (7.42%). Children with ADHD (versus no ADHD) had higher odds of anxiety/depression. Engaging in PA (versus no PA) and having immigrant parents (versus non-immigrant parents) were associated with lower anxiety/depression odds. ADHD diagnosis status significantly moderated the association between anxiety/depression and PA or parental nativity. However, the three-way interaction between ADHD status, parental nativity, and physical activity was not statistically significant. Stratified by ADHD diagnosis status, those who engaged in PA (versus did not) for 1 to 3 days, 4 to 6 days, and daily were less often diagnosed with anxiety/depression disorder among those with or without ADHD, especially children without ADHD. The odds were also lower for children with or without ADHD whose parents were immigrants than children with non-immigrant parents, particularly children without ADHD who had immigrant parents. Parental nativity did not significantly moderate the association between PA and anxiety/depression among children with and without ADHD.

**Conclusions:**

Physical activity was associated with lower risks of diagnosed with anxiety/depression disorder among children, especially children without ADHD and those with immigrant parents. Considering parental nativity and incorporating personalized PA in ADHD and anxiety/depression management can improve mental illness and ADHD symptoms among children.

## Background

Attention-deficit/hyperactivity disorder (ADHD) is a neurodevelopmental disorder characterized by a range of behavioral symptoms, including difficulty in sustaining attention, hyperactivity, and impulsive behavior, which often leads to functional impairments in various aspects of life [1, 2]. Children diagnosed with ADHD face higher risks of encountering anxiety and depression, which can magnify their primary disorder’s burden [3–5]. Childhood anxiety and depression can result in academic struggles, social difficulties, higher chances of substance misuse, and suicidal thoughts, impacting a person’s life path [6–8]. Anxiety and depression, among the most common, co-occurring, and sequential mental health disorder symptoms that are often measured together as general psychological distress or mental health in children and adults, have elevated risks for disability severity [9–21]. ADHD and anxiety/depression intersect as significant mental health issues, compounding their effects and significantly harming children’s quality of life and future prospects [22]. These conditions together elevate children’s daily challenges, underscoring the need for effective and comprehensive interventions to relieve their compound burden.

Physical activity and parental background are associated with anxiety/depression and ADHD in children. Physical activity has known mental health benefits, including reducing symptoms of anxiety/depression and ADHD with minimal side effects [23–27], unlike pharmacotherapy that can result in significant side effects [28–35]. It is frequently used to enhance cognitive and emotional states, leading to better moods, increased concentration, and reduced behavioral problems [23, 36, 37]. Parental nativity can play a crucial role in shaping disparities in the occurrence and impact of anxiety/depression and ADHD within this complex interplay. Children of immigrant parents often exhibit better mental health and behavioral outcomes than their native-born counterparts [38–40], but the impact of parental nativity on anxiety/depression in children with ADHD has not been investigated. Exploring the synergy of physical activity and parental nativity can reveal nuanced insights into anxiety/depression patterns in children with or without ADHD.

Children’s mental health, especially in those with ADHD, is intricately connected to environmental and sociodemographic factors that need to be accounted for in studies examining children’s mental health. Studies emphasize the significant impact of neighborhood safety, as exposure to unsafe environments correlates with increased anxiety/depressive disorders in children [38, 41–43]. Supportive networks and social capital are recognized in mitigating mental health issues, emphasizing the importance of community cohesion and trust [41, 44, 45]. Contemporary research emphasizes inclusive spaces and nature-based interventions to enhance mental resilience in children with ADHD [46, 47]. Age, gender, and race/ethnicity affect mental health, with males, older individuals, and racial/ethnic minority children at greater risk of disorders like ADHD and anxiety/depression compared to females, younger individuals, and non-racial/ethnic minorities [1, 48]. Other sociodemographic factors, such as parental unemployment and lower educational attainment, have also been associated with a higher risk of ADHD and anxiety/depression in children [1, 38, 49].

The multifactorial interplay between ADHD, anxiety/depression, physical activity, parental nativity, and environmental factors necessitates an in-depth exploration. The insights from this exploration can play a crucial role in developing effective non-pharmacological interventions to address mental health among children. The current study explored the association between ADHD diagnosis status, physical activity, parental nativity, and anxiety/depression among children. We also assessed whether ADHD diagnosis status moderates the association between physical activity or parental nativity and anxiety/depression among children. Furthermore, we examined the association between physical activity, parental nativity, and anxiety/depression among children with and without ADHD. Additionally, we examined whether parental nativity moderates the association between physical activity and anxiety/depression among children with and without ADHD.

## Methods

### Study setting and design

We conducted a secondary data analysis of the 2016–2021 National Survey of Children’s Health (NSCH), annual online and mail cross-sectional surveys. The NSCH data are de-identified and publicly available. NSCH is a nationally representative survey of noninstitutionalized children aged 0–17 years selected from the U.S. 50 states and the District of Columbia [50]. NSCH provides national and state-level estimates of children’s physical and mental (e.g., anxiety, depression, ADHD) health, health behaviors (e.g., physical activity), their families and communities, as well as neighborhood characteristics. NSCH is sponsored by the Health Resources and Services Administration’s Maternal and Child Health Bureau (HRSA MCHB) within the U.S. Department of Health and Human Services. HRSA MCHB contracts with the U.S. Census Bureau conducted the survey. The sampling involves selecting households with children from each state and the District of Columbia. A parent or caregiver of the selected child completes the surveys. The total pooled data from the 2016 to 2021 surveys is 225,443. We restricted the analysis to children aged 6–17 years (*n* = 155,178); only these age groups were eligible for the physical activity questions. Of the total 155,178 children, 14,201 of them had missing observations on all the analytical variables, resulting in analytical sample of 140,977 children with complete observations on all the analytical variables. We therefore performed complete case analysis on the 140,977 samples because the missingness on the selected variables ranged from 0.20 to 3.30%, which are less than the *≥* 5% or *≥* 10% missingness thresholds for biased estimates. We did not include the NSCH data before 2016 because the prior surveys were not conducted annually. This study followed the Strengthening the Reporting of Observational Studies in Epidemiology (STROBE) reporting guideline [51].

### Outcome

Current anxiety/depression among children was derived from two questions. The parents/caregivers were asked two questions to be eligible for the follow-up questions. Firstly, the parents/caregivers of the children were asked (Yes/No), “Has a doctor or other health care provider ever told you that this child has…Depression?” Next, they were asked (Yes/No), “Has a doctor or other health care provider ever told you this child has…Anxiety Problems? The two follow-up questions (Yes/No) to determine current anxiety or depression include “If yes, does this child currently have the condition?” We recategorized the responses into current anxiety/depression if the children currently have either anxiety or depression, or they have both. They have no current anxiety/depression if they never had anxiety or depression, or if they ever had but currently do not have anxiety or depression.

### Exposures

The main predictors included a child’s physical activity status, parental nativity, and ADHD status. For the child’s physical activity status, the children’s parents/caregivers were asked to report on how many days (0 days, 1 to 3 days, 4 to 6 days, or every day) their child exercised, played a sport, or participated in physical activity for at least 60 min during the past week. Parental nativity was reported based on the question, “What is this child’s parent(s) generational status? The responses were coded as non-immigrant (Parents born in the U.S.), immigrant (Parents born outside of the U.S.), or other (parents unknown or not listed). The children’s ADHD status was reported by their parents/caregivers with the question (Yes/No), “Has a doctor or other health care provider ever told you that this child has… attention deficit disorder or attention-deficit/hyperactivity disorder, that is, ADD or ADHD?” Similar to previous national studies that analyzed ever diagnosis of ADHD [52–55], we included ADHD diagnosis status in this study based on ever diagnosis status.

### Covariates

We adjusted for the children’s sociodemographic characteristics, including age, sex, race/ethnicity, educational level of adults in the child’s household, and nativity (Born in the U.S. or born outside the U.S.). The neighborhood characteristics (i.e., safety, support/cohesion/social capital, amenities, and detracting elements) were also analyzed. Neighborhood safety was assessed with the question (safe or unsafe neighborhood), “Does this child live in a safe neighborhood? Neighborhood support/cohesion/social capital was measured with three questions on a 4-point Likert scale, with total scores ranging from 3 to 12; higher scores indicate a higher neighborhood support/cohesion/social capital [56–58].

Neighborhood amenities were assessed with four questions (Yes/No) based on four items: parks/playgrounds, recreation centers/community center/boys’ and girls’ clubs, sidewalks/walking paths, and libraries/bookmobiles. For each of the four items, the children’s parents/caregivers were asked whether their child lives in a neighborhood with those four amenities. Neighborhood detracting elements were also determined with four questions (Yes/No) based on three items: litter/garbage on the street/sidewalk, poorly kept/rundown housing, and vandalism such as broken windows/graffiti. The parents/caregivers responded to questions about whether this child lives in a neighborhood with any of the three items.

### Statistical analysis

**Fig. 1:**
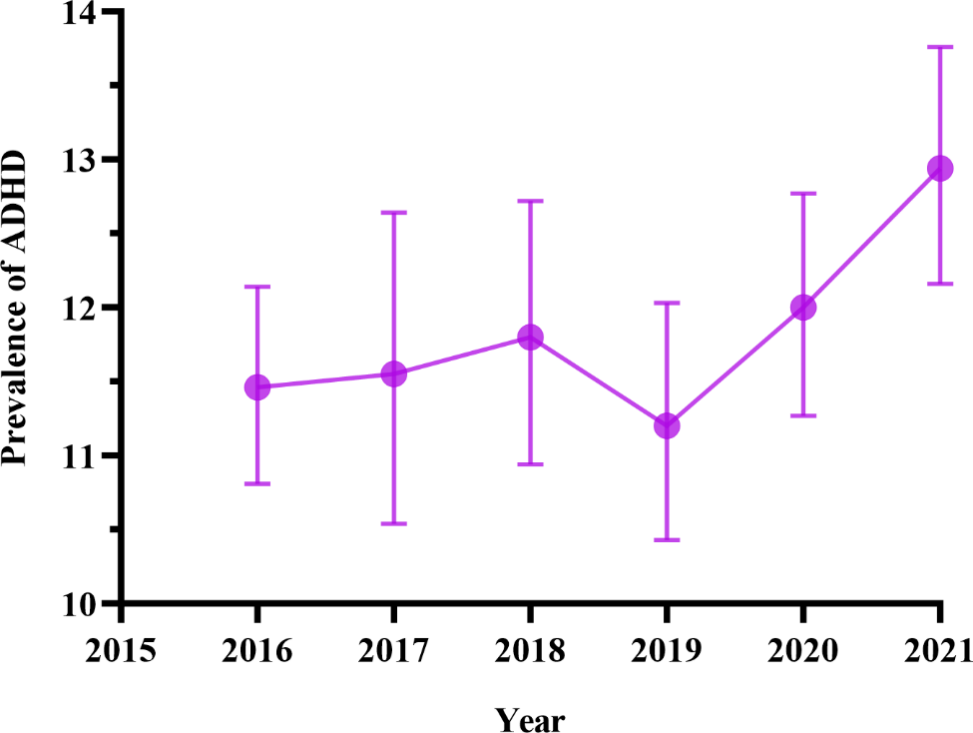
Trends of ADHD prevalence among children aged 6–17 years

**Fig. 2:**
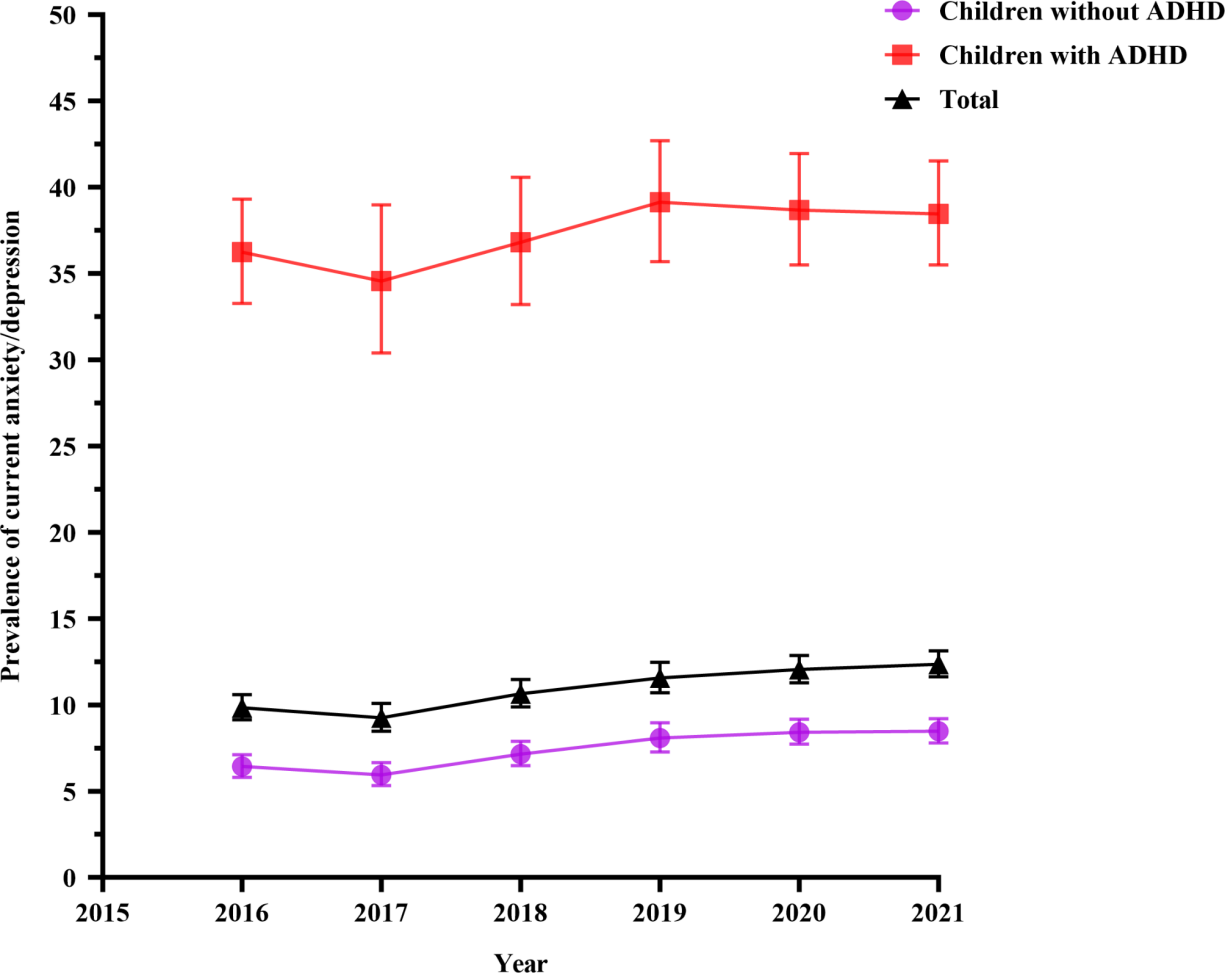
Trends of current anxiety/depression prevalence among children and those with and without ADHD diagnosis

**Fig. 3:**
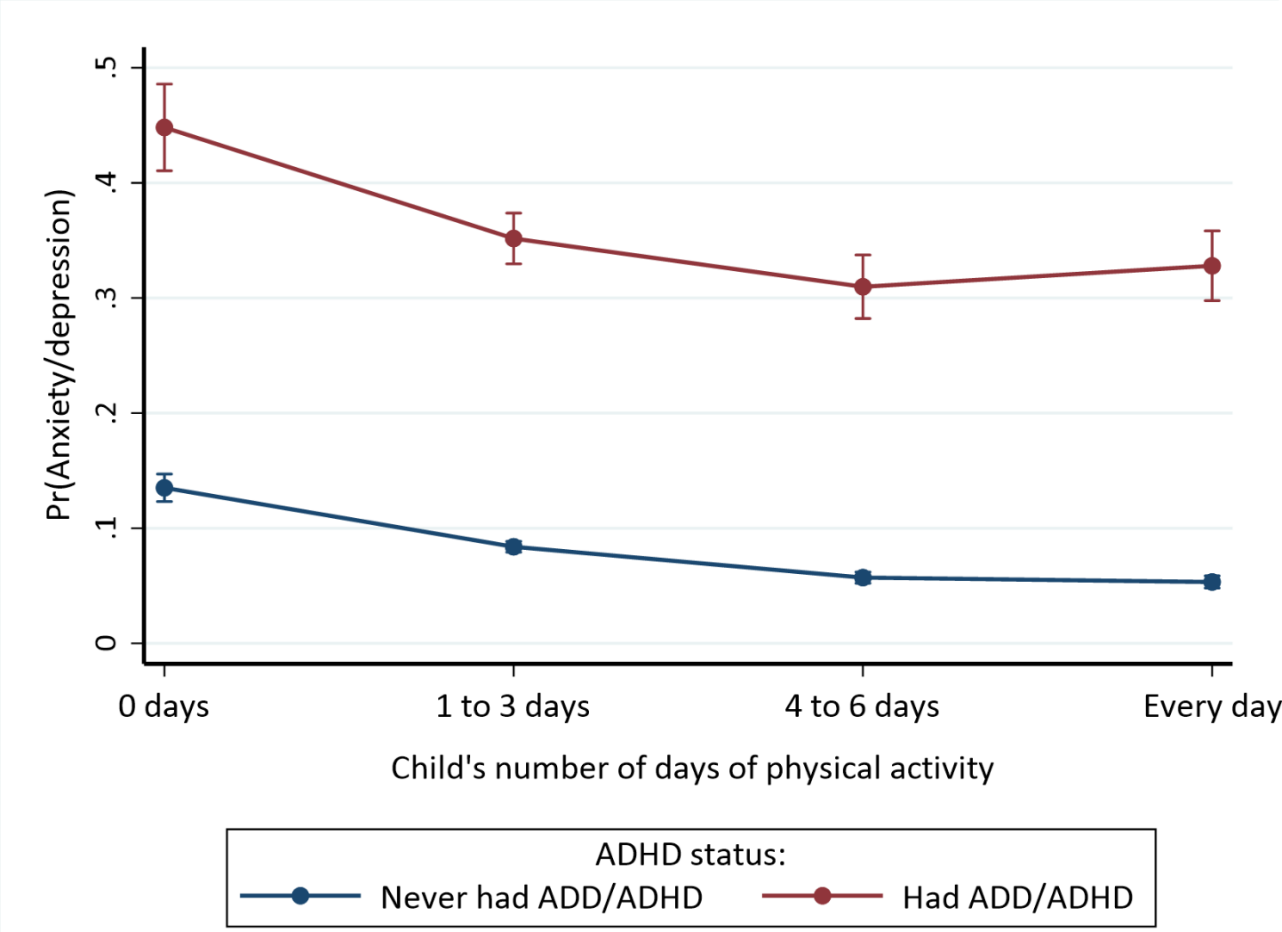
Moderation effect of ADHD diagnosis status on the association between current anxiety/depression and number of days of physical activity among children, adjusting for sociodemographic and neighborhood characteristics

**Fig. 4:**
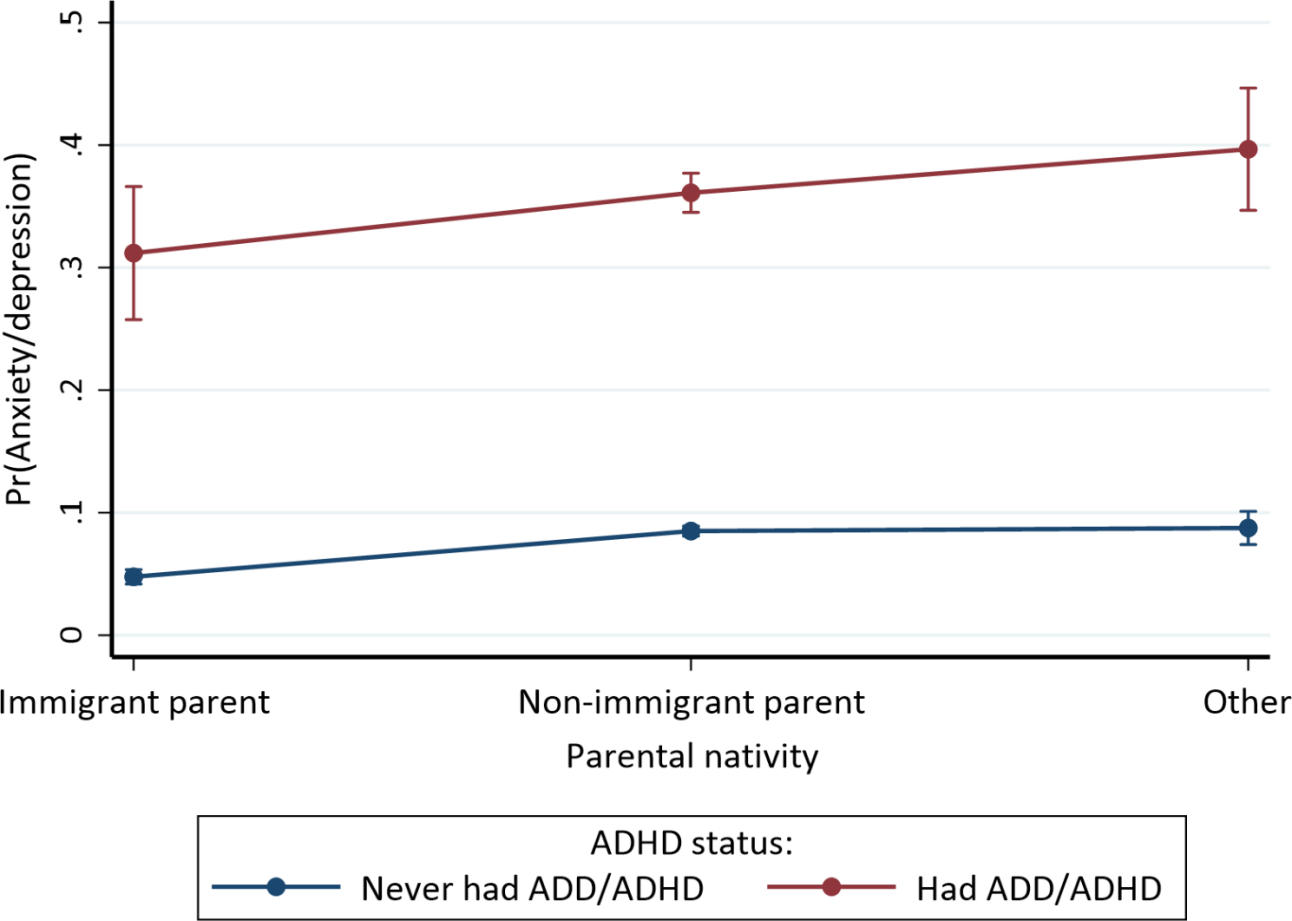
Moderation effect of ADHD diagnosis status on the association between current anxiety/depression and parental nativity among children, adjusting for sociodemographic and neighborhood characteristics. Other means parent unknown

Weighted analysis using the NSCH complex survey weight and nesting variables (strata [states of residence and sampling stratum] and primary sampling unit [household]) to provide nationally representative estimates that reflect noninstitutionalized child aged 6–17 years population in the U.S. First, we estimated the trends in the prevalence of ADHD from 2016 to 2021 (Fig. 1). Next, we computed the trends in the prevalence of current anxiety/depression from 2016 to 2021 based on ADHD status (Fig. 2). For the following analyses, we calibrated (weight divided by the number of survey years) the weight to compute average estimates across the six survey years of data. Third, we used chi-square tests (i.e., Rao-Scott chi-square tests) and t-test to assess differences in current anxiety/depression by the main predictors (physical activity status, parental nativity, and ADHD diagnosis status) and the covariates (sociodemographic and neighborhood characteristics), and stratified by ADHD diagnosis status (Table 1). Fourth, we used six multivariable logistic regression models to examine the associations between current anxiety/depression and the predictors, as well as the interactions between the predictors, adjusting for the covariates (Table 2). For the statistically significant interactions, we evaluated interaction effects and estimated the average predicted probabilities or marginal effects to determine whether ADHD diagnosis status moderates the association between current anxiety/depression and physical activity (Fig. 3) and parental nativity (Fig. 4), adjusting for the covariates. Additionally, we conducted multivariable logistic regression analysis to assess the association between current anxiety/depression and physical activity and parental nativity as well as their interaction among children with and without ADHD, adjusting for the covariates (Table 3). We performed pairwise or multiple comparisons tests using the Bonferroni method to compare current anxiety/depression across physical activity levels or parental nativity groups as well as significant interactions. The comparisons tests using the Bonferroni method help to determine which groups are significantly different from each other and to reduce Type 1 error in multiple hypothesis tests [59]. All analyses were performed using STATA version 18.0. We estimated adjusted odds ratio (AOR) with 95% confidence intervals (CIs). Statistical significance levels were estimated at *p* < 0.05 with 2-sided tests.

**Table 1:** Descriptive and bivariate analyses of the current anxiety/depression by physical activity status, parental nativity, sociodemographic factors, and neighborhood characteristics among U.S. children (*N* = 140,977) and those with (*n* = 19,082) and without (*n* = 121,895) ADHD

	All children		*p*-value	Children without ADHD		*p*-value	Children with ADHD		*p*-value
	Total sample	Current anxiety/depression prevalence		Overall sample without ADHD	Current anxiety/depression prevalence		Overall sample with ADHD	Current anxiety/depression prevalence	
	% (95% CI)	% (95% CI)		% (95% CI)	% (95% CI)		% (95% CI)	% (95% CI)	
Overall		10.96 (10.64, 11.29)			7.42 (7.13, 7.72)			37.34 (35.93, 38.77)	
ADHD diagnosis status			< 0.001						
No ADHD	88.17 (87.83, 88.51)	7.42 (7.13, 7.72)		–	–		–	–	
ADHD	11.83 (11.49, 12.17)	37.34 (35.93, 38.77)		–	–		–	–	
Child’s number of days of physical activity			< 0.001			< 0.001			< 0.001
0 days	10.00 (9.63, 10.38)	20.36 (19.00, 21.79)		9.48 (9.09, 9.90)	14.20 (12.90, 15.61)		13.83 (12.85, 14.89)	51.83 (47.87, 55.77)	
1 to 3 days	40.67 (40.08, 41.27)	11.88 (11.36, 12.43)		40.60 (39.96, 41.25)	8.35 (7.88, 8.86)		41.18 (39.71, 42.66)	37.84 (35.65, 40.09)	
4 to 6 days	27.38 (26.87, 27.89)	8.70 (8.16, 9.28)		27.78 (27.23, 28.34)	5.86 (5.38, 6.38)		24.39 (23.09, 25.73)	32.85 (30.09, 35.75)	
Every day	21.95 (21.46, 22.45)	7.77 (7.22, 8.36)		22.14 (21.60, 22.68)	4.75 (4.30, 5.26)		20.60 (19.42, 21.84)	31.91 ([29.00, 34.97)	
Parental nativity			< 0.001			< 0.001			0.091
Immigrant	27.01 (26.39, 27.63)	5.89 (5.28, 6.55)		29.01 (28.34, 29.69)	4.41 (3.88, 5.00)		12.09 [10.85, 13.46)	32.23 (26.95, 38.01)	
Non-immigrant	67.63 [67.00, 68.26)	12.77 (12.39, 13.16)		66.10 (65.41, 66.78)	8.71 (8.36, 9.07)		79.07 (77.59, 80.47)	38.10 (36.61, 39.61)	
Other (Parent unknown)	5.36 (5.06, 5.67)	13.66 (12.26, 15.19)		4.89 (4.58, 5.23)	7.88 (6.73, 9.21)		8.84 (8.02, 9.74)	37.51 (32.96, 42.30)	
Child’s age (in years)			< 0.001			< 0.001			< 0.001
6–11	49.62 (49.02, 50.22)	7.66 (7.27, 8.08)		50.59 (49.94, 51.24)	4.75 (4.42, 5.10)		42.36 (40.87, 43.87)	33.61 (31.43, 35.87)	
12–17	50.38 (49.78, 50.98)	14.20 (13.71, 14.71)		49.41 (48.76, 50.06)	10.15 (9.69, 10.64)		57.64 (56.13, 59.13)	40.08 (38.24, 41.94)	
Child’s sex			< 0.001			< 0.001			< 0.001
Male	51.10 (50.50, 51.70)	10.14 (9.72, 10.58)		48.76 (48.11, 49.41)	5.59 (5.25, 5.95)		68.50 (67.04, 69.93)	34.31 (32.64, 36.01)	
Female	48.90 (48.30, 49.50)	11.81 (11.33, 12.30)		51.24 (50.59, 51.89)	9.16 (8.70, 9.64)		31.50 (30.08, 32.96)	43.93 (41.23, 46.66)	
Child’s race/ethnicity			< 0.001			< 0.001			< 0.001
Hispanic	25.25 (24.60, 25.91)	8.77 (7.96, 9.65)		26.13 (25.42, 26.85)	6.31 (5.57, 7.14)		18.73 (17.21, 20.34)	34.34 (29.97, 38.98)	
Non-Hispanic Asian	4.52 (4.31, 4.74)	3.20 (2.58, 3.95)		4.99 (4.76, 5.24)	2.68 (2.08, 3.45)		0.99 (0.81, 1.20)	22.52 (16.94, 29.29)	
Non-Hispanic Black	13.02 (12.60, 13.45)	8.33 (7.46, 9.29)		12.69 (12.24, 13.15)	4.55 (3.88, 5.34)		15.44 (14.22, 16.75)	31.51 (27.47, 35.84)	
Other/Multi-racial, non-Hispanic	5.91 (5.69, 6.14)	12.69 (11.46, 14.04)		5.86 (5.62, 6.11)	8.15 (7.03, 9.42)		6.29 (5.72, 6.92)	44.26 (39.50, 49.14)	
Non-Hispanic White	51.30 (50.71, 51.89)	13.19 (12.81, 13.57)		50.33 (49.70, 50.96)	9.10 (8.77, 9.45)		58.55 (56.95, 60.14)	39.34 (37.86, 40.85)	
Child’s nativity			< 0.001			< 0.001			0.572
Born in the U.S.	95.08 (94.79, 95.35)	11.17 (10.84, 11.50)		94.81 (94.50, 95.11)	7.58 (7.29, 7.89)		97.08 (96.33, 97.69)	37.25 (35.83, 38.69)	
Born outside U.S.	4.92 (4.65, 5.21)	6.92 (5.57, 8.57)		5.19 (4.89, 5.50)	4.39 (3.27, 5.88)		2.92 (2.32, 3.67)	40.45 (29.93, 51.92)	
Educational level of adults in child’s household			< 0.001			< 0.001			0.173
Less than high school	9.66 (9.11, 10.23)	7.59 (6.36, 9.04)		9.96 (9.37, 10.59)	5.30 (4.16, 6.74)		7.35 (6.17, 8.73)	30.72 (23.99, 38.39)	
High school or GED	19.47 (18.96, 19.98)	10.70 (9.88, 11.57)		19.22 (18.67, 19.79)	6.76 (6.06, 7.53)		21.27 (19.99, 22.61)	37.23 (33.78, 40.80)	
Some college/technical school	21.68 (21.22, 22.15)	12.18 (11.54, 12.86)		21.09 (20.60, 21.60)	7.92 (7.36, 8.51)		26.03 (24.79, 27.31)	37.97 (35.37, 40.65)	
College degree or higher	49.20 (48.61, 49.80)	11.18 (10.78, 11.59)		49.72 (49.07, 50.37)	7.89 (7.52, 8.27)		45.35 (43.89, 46.81)	38.10 (36.31, 39.92)	
Neighborhood safety			< 0.001			< 0.001			< 0.001
Unsafe neighborhood	5.18 (4.86, 5.52)	18.59 (16.28, 21.14)		5.01 (4.66, 5.38)	13.26 (10.99, 15.92)		6.43 (5.67, 7.28)	49.53 (43.07, 56.02)	
Safe neighborhood	94.82 (94.49, 95.14)	10.54 (10.23, 10.86)		94.99 (94.62, 95.34)	7.11 (6.84, 7.40)		93.57 (92.72, 94.33)	36.50 (35.07, 37.96)	
Neighborhood support/cohesion/social capital (Mean [SD] 95% CI)	9.69 ([2.127] 9.66, 9.72)	9.20 ([2.620] 9.12, 9.29)	< 0.001	9.74 ([2.082] 9.71, 9.77)	9.27 ([2.595] 9.16, 9.38)	< 0.001	9.37 ([2.438] 9.30, 9.44)	9.10 ([2.652] 8.98, 9.23)	< 0.001
Neighborhood amenities			0.921			0.965			0.089
None	10.71 (10.39, 11.04)	11.00 (10.12, 11.94)		10.53 (10.18, 10.89)	7.40 (6.60, 8.29)		12.09 (11.25, 12.97)	34.38 (30.98, 37.95)	
Neighborhood contains 1 to 4 amenities	89.29 (88.96, 89.61)	10.95 (10.61, 11.30)		89.47 (89.11, 89.82)	7.42 (7.11, 7.74)		87.91 (87.03, 88.75)	37.75 (36.21, 39.31)	
Neighborhood detracting elements			< 0.001			< 0.001			0.028
None	74.57 (73.99, 75.14)	10.38 (10.05, 10.72)		74.84 (74.21, 75.45)	7.01 (6.72, 7.31)		72.61 (71.10, 74.07)	36.26 (34.73, 37.81)	
Neighborhood has all 3 detracting elements	25.43 (24.86, 26.01)	12.66 (11.87, 13.50)		25.17 (24.55, 25.79)	8.64 (7.91, 9.44)		27.39 (25.93, 28.91)	40.21 (37.04, 43.47)	

Percentages were weighted

*SD* standard deviation

**Table 2:** Adjusted multivariable logistic regression analysis of current anxiety/depression with physical activity status, parental nativity, sociodemographic factors, and neighborhood characteristics among U.S. children (*n* = 140,977)

	Model 1	Model 2	Model 3	Model 4	Model 5	Model 6
	AOR (95% CI)	AOR (95% CI)	AOR (95% CI)	AOR (95% CI)	AOR (95% CI)	AOR (95% CI)
ADHD diagnosis status						
No ADHD	1 [Reference]	1 [Reference]	1 [Reference]	1 [Reference]	1 [Reference]	1 [Reference]
ADHD	6.70*** (6.20, 7.24)	7.23*** (6.67, 7.84)	5.82*** (4.78, 7.07)	5.81*** (6.26, 7.41)	7.24*** (6.68, 7.85)	5.17*** (4.20, 6.35)
Child’s number of days of physical activity						
0 days	1 [Reference]	1 [Reference]	1 [Reference]	1 [Reference]	1 [Reference]	1 [Reference]
1 to 3 days	0.54*** (0.49, 0.60)	0.59*** (0.53, 0.66)	0.58*** (0.51, 0.65)	0.60*** (0.53, 0.66)	0.60*** (0.54, 0.67)	0.57*** (0.50, 0.65)
4 to 6 days	0.37*** (0.33, 0.41)	0.42*** (0.37, 0.47)	0.38*** (0.33, 0.43)	0.42*** (0.37, 0.47)	0.44*** (0.38, 0.50)	0.39*** (0.33, 0.45)
Every day	0.31*** (0.28, 0.35)	0.41*** (0.36, 0.47)	0.35*** (0.30, 0.41)	0.41*** (0.36, 0.47)	0.42*** (0.36, 0.48)	0.35*** (0.30, 0.41)
Parental nativity						
Immigrant	0.49*** (0.43, 0.55)	0.58*** (0.51, 0.67)	0.58*** (0.51, 0.66)	0.53*** (0.45, 0.62)	0.67** (0.51, 0.88)	0.58** (0.42, 0.79)
Non-immigrant	1 [Reference]	1 [Reference]	1 [Reference]	1 [Reference]	1 [Reference]	1 [Reference]
Other (Parent unknown)	0.86* (0.75, 0.99)	1.09 (0.94, 1.26)	1.09 (0.94, 1.26)	1.03 (0.86, 1.24)	0.96 (0.71, 1.29)	0.79 (0.55, 1.14)
ADHD diagnosis status X Child’s number of days of physical activity			F (3, 140368) = 6.28, *p* < 0.001			
ADHD X 0 days			1 [Reference]			
ADHD X 1 to 3 days			1.12 (0.89, 1.41)			
ADHD X 4 to 6 days			1.40* (1.08, 1.80)			
ADHD X Every day			1.65*** (1.26, 2.15)			
ADHD diagnosis status X parental nativity				F (2, 140369) = 3.27, *p*= 0.038		
ADHD X non-immigrant				1 [Reference]		
ADHD X immigrant				1.49* (1.08, 2.04)		
ADHD X Other (Parent unknown)				1.14 (0.85, 1.54)		
Parental nativity X Child’s number of days of physical activity					F (6, 140365) = 1.06, *p* = 0.387	
Non-immigrant X 0 days					1 [Reference]	
Immigrant X 1 to 3 days					0.90 (0.65, 1.25)	
Other (Parent unknown) X 1 to 3 days					1.17 (0.81, 1.70)	
Immigrant X 4 to 6 days					0.70 (0.48, 1.00)	
Other (Parent unknown) X 4 to 6 days					1.08 (0.71, 1.66)	
Immigrant X Every day					0.84 (0.54, 1.30)	
Other (Parent unknown) X Every day					1.31 (0.86, 2.00)	
ADHD X Parental nativity X Child’s number of days of physical activity						F (6, 140365) = 0.56, *p* = 0.763
No ADHD X Non-immigrant X 0 days						1 [Reference]
ADHD X Immigrant X 1 to 3 days						0.92 (0.40, 2.10)
ADHD X Other (Parent unknown) X 1 to 3 days						0.51 (0.23, 1.13)
ADHD X Immigrant X 4 to 6 days						0.94 (0.38, 2.32)
ADHD X Other (Parent unknown) X 4 to 6 days						0.83 (0.34, 2.04)
ADHD X Immigrant X Every day						1.02 (0.36, 2.87)
ADHD X Other (Parent unknown) X Every day						0.61 (0.25, 1.51)
Child’s age (in years)						
6–11		1 [Reference]	1 [Reference]	1 [Reference]	1 [Reference]	1 [Reference]
12–17		1.81*** (1.68, 1.95)	1.81*** (1.68 1.95)	1.81*** (1.68, 1.95)	1.81*** (1.68, 1.95)	1.81*** (1.68, 1.95)
Child’s sex						
Male		1 [Reference]	1 [Reference]	1 [Reference]	1 [Reference]	1 [Reference]
Female		1.58*** (1.47, 1.70)	1.57*** (1.46, 1.69)	1.58***(1.47, 1.70)	1.58***(1.47, 1.70)	1.57***(1.46, 1.69)
Child’s Race and Ethnicity						
Hispanic		0.77** (0.67, 0.87)	0.76*** (0.67, 0.87)	0.77*** (0.68, 0.88)	0.77*** (0.67, 0.87)	0.77*** (0.67, 0.88)
Non-Hispanic Asian		0.36*** (0.28, 0.47)	0.36*** (0.28, 0.46)	0.38*** (0.29, 0.48)	0.36*** (0.28, 0.47)	0.37*** (0.29, 0.48)
Non-Hispanic Black		0.45*** (0.39, 0.52)	0.45*** (0.39, 0.51)	0.45*** (0.40, 0.52)	0.45*** (0.39, 0.52)	0.45*** (0.40, 0.52)
Non-Hispanic White		1 [Reference]	1 [Reference]	1 [Reference]	1 [Reference]	1 [Reference]
Other/Multi-racial, non-Hispanic		0.97 (0.85, 1.11)	0.97 (0.85, 1.11)	0.97 (0.85, 1.11)	0.97 (0.85, 1.11)	0.98 (0.86, 1.12)
Child’s nativity						
Born in the U.S.		1 [Reference]	1 [Reference]	1 [Reference]	1 [Reference]	1 [Reference]
Born outside U.S.		0.92 (0.72, 1.19)	0.92 (0.71, 1.19)	0.93 (0.72, 1.21)	0.91 (0.71, 1.18)	0.92 (0.72, 1.19)
Educational level of adults in child’s household						
Less than high school		1 [Reference]	1 [Reference]	1 [Reference]	1 [Reference]	1 [Reference]
High school or GED		1.21 (0.96, 1.53)	1.21 (0.97, 1.53)	1.20 (0.95, 1.51)	1.22 (0.97, 1.54)	1.21 (0.96, 1.52)
Some college/technical school		1.38** (1.11, 1.72)	1.38** (1.11, 1.72)	1.36** (1.09, 1.70)	1.39** (1.16, 1.73)	1.37** (1.10, 1.71)
College degree or higher		1.52*** (1.22, 1.88)	1.52*** (1.23, 1.89)	1.49*** (1.20, 1.86)	1.52*** (1.23, 1.90)	1.51*** (1.21, 1.87)
Neighborhood safety						
Unsafe neighborhood		1 [Reference]	1 [Reference]	1 [Reference]	1 [Reference]	1 [Reference]
Safe neighborhood		0.65*** (0.54, 0.80)	0.65*** (0.53, 0.79)	0.65*** (0.54, 0.80)	0.65*** (0.54, 0.80)	0.65*** (0.53, 0.79)
Neighborhood support/cohesion/social capital (Mean [SD])		0.91*** (0.89, 0.93)	0.91*** (0.89, 0.93)	0.91*** (0.89, 0.93)	0.91*** (0.89, 0.93)	0.91*** (0.89, 0.93)
Neighborhood amenities						
None		1 [Reference]	1 [Reference]	1 [Reference]	1 [Reference]	1 [Reference]
Neighborhood contains 1 to 4 amenities		1.22** (1.09, 1.36)	1.22** (1.09, 1.37)	1.22** (1.09, 1.36)	1.22** (1.09, 1.36)	1.22** (1.09, 1.36)
Neighborhood detracting elements						
None		1 [Reference]	1 [Reference]	1 [Reference]	1 [Reference]	1 [Reference]
Neighborhood has all 3 detracting elements		1.16** (1.06, 1.28)	1.16** (1.06, 1.27)	1.16** (1.06, 1.27)	1.16** (1.06, 1.28)	1.16** (1.05, 1.27)

*AOR* adjusted odds ratio, *95% CI* 95% confidence interval. Statistical significance at *p < 0.05, **p < 0.01, and ***p < 0.001

**Table 3:** Adjusted multivariable logistic regression analysis of current anxiety/depression with physical activity status, parental nativity, sociodemographic factors, and neighborhood characteristics among U.S. children with (*n* = 19,082) and without (*n* = 121,895) ADHD

	Children without ADHD		Children with ADHD	
	Model 1	Model 2	Model 3	Model 4
	Anxiety/depression		Anxiety/depression	
	AOR (95% CI)	AOR (95% CI)	AOR (95% CI)	AOR (95% CI)
Child’s number of days of physical activity				
0 days	1 [Reference]	1 [Reference]	1 [Reference]	1 [Reference]
1 to 3 days	0.58*** (0.51, 0.66)	0.58*** (0.51, 0.66)	0.61*** (0.51, 0.74)	0.64*** (0.52, 0.77)
4 to 6 days	0.38*** (0.33, 0.43)	0.39*** (0.34, 0.46)	0.50*** (0.40, 0.61)	0.52*** (0.42, 0.65)
Every day	0.36*** (0.31, 0.42)	0.36*** (0.31, 0.43)	0.51*** (0.41, 0.64)	0.52*** (0.41, 0.66)
Parental nativity				
Immigrant	0.53*** (0.45, 0.62)	0.60** (0.43, 0.82)	0.79 (0.60, 1.04)	0.88 (0.50, 1.55)
Non-immigrant	1 [Reference]	1 [Reference]	1 [Reference]	1 [Reference]
Other (Parent unknown)	1.07 (0.88, 1.29)	0.83 (0.57, 1.20)	1.10 (0.88, 1.37)	1.27 (0.76, 2.14)
Parental nativity X Child’s number of days of physical activity		F (6, 140365) = 1.33, *p* = 0.239		F (6, 140348) = 0.37, *p* = 0.896
Non-immigrant X 0 days		1 [Reference]		1 [Reference]
Immigrant X 1 to 3 days		0.93 (0.64, 1.35)		0.93 (0.46, 1.88)
Other (Parent unknown) X 1 to 3 days		1.47 (0.93, 2.32)		0.75 (0.40, 1.41)
Immigrant X 4 to 6 days		0.69 (0.46, 1.04)		0.71 (0.33, 1.51)
Other (Parent unknown) X 4 to 6 days		1.09 (0.64, 1.84)		0.92 (0.45, 1.88)
Immigrant X Every day		0.86 (0.51, 1.46)		1.02 (0.43, 2.41)
Other (Parent unknown) X Every day		1.49 (0.84, 2.64)		0.94 (0.47, 1.85)
Child’s age (in years)				
6–11	1 [Reference]	1 [Reference]	1 [Reference]	1 [Reference]
12–17	2.14*** (1.95, 2.35)	2.14*** (1.95, 2.35)	1.26** (1.10, 1.43)	1.26** (1.10, 1.43)
Child’s sex				
Male	1 [Reference]	1 [Reference]	1 [Reference]	1 [Reference]
Female	1.61*** (1.48, 1.77)	1.61*** (1.48, 1.76)	1.48***(1.30, 1.69)	1.48*** (1.30, 1.69)
Child’s Race and Ethnicity				
Hispanic	0.77** (0.65, 0.90)	0.77***	0.76* (0.61, 0.94)	0.76* (0.61, 0.94)
Non-Hispanic Asian	0.36*** (0.27, 0.48)	0.36*** (0.27, 0.48)	0.41*** (0.27, 0.63)	0.41*** (0.26, 0.62)
Non-Hispanic Black	0.37*** (0.31, 0.44)	0.37*** (0.31, 0.44)	0.62*** (0.50, 0.77)	0.62*** (0.50, 0.77)
Non-Hispanic White	1 [Reference]	1 [Reference]	1 [Reference]	1 [Reference]
Other/Multi-racial, non-Hispanic	0.89 (0.75, 1.60)	0.90 (0.76, 1.07)	1.16 (0.93, 1.44)	1.17 (0.94, 1.46)
Child’s nativity				
Born in the U.S.	1 [Reference]	1 [Reference]	1 [Reference]	1 [Reference]
Born outside U.S.	0.81 (0.59, 1.12)	0.80 (0.58, 1.10)	1.34 (0.85, 2.13)	1.37 (0.87, 2.14)
Educational level of adults in child’s household				
Less than high school	1 [Reference]	1 [Reference]	1 [Reference]	1 [Reference]
High school or GED	1.17 (0.88, 1.56)	1.18 (0.89, 1.58)	1.35 (0.91, 1.99)	1.35 (0.91, 1.99)
Some college/technical school	1.38* (1.05, 1.81)	1.39* (1.06, 1.83)	1.43 (0.98, 2.07)	1.43 (0.98, 2.08)
College degree or higher	1.56** (1.19, 2.04)	1.58** (1.20, 2.06)	1.48* (1.03, 2.15)	1.48* (1.03, 2.15)
Neighborhood safety				
Unsafe neighborhood	1 [Reference]	1 [Reference]	1 [Reference]	1 [Reference]
Safe neighborhood	0.63*** (0.50, 0.80)	0.63*** (0.50, 0.80)	0.74 (0.54, 1.02)	0.73 (0.53, 1.01)
Neighborhood support/cohesion/social capital (Mean [SD])	0.90*** (0.88, 0.92)	0.90*** (0.88, 0.92)	0.93*** (0.89, 0.96)	0.93*** (0.90, 0.96)
Neighborhood amenities				
None	1 [Reference]	1 [Reference]	1 [Reference]	1 [Reference]
Neighborhood contains 1 to 4 amenities	1.19* (1.03, 1.37)	1.19* (1.03, 1.37)	1.28** (1.08, 1.53)	1.29** (1.08, 1.53)
Neighborhood detracting elements				
None	1 [Reference]	1 [Reference]	1 [Reference]	1 [Reference]
Neighborhood has all 3 detracting elements	1.18** (1.05, 1.32)	1.18** (1.05, 1.32)	1.11 (0.95, 1.31)	1.11 (0.94, 1.31)

*AOR* adjusted odds ratio, *95% CI* 95% confidence interval

Statistical significance at *p < 0.05, **p < 0.01, and ***p < 0.001

## Results

### Descriptive characteristics

Among all the children aged 6–17 years included in the study, the weighted prevalence of current anxiety/depression diagnosis was 10.96% (Table 1). The prevalence of ADHD diagnosis was 11.83%. Higher proportions of the children engaged in physical activity for 1 to 3 days (40.67%), had non-immigrant parents (67.63%), aged 12–17 years (50.38%), were males (51.10%), non-Hispanic White individuals (51.30%), born in the U.S. (95.08%), lived in household with adults with college or higher degree (49.20%), lived in a safe neighborhood (94.82%), or lived in neighborhood without any detracting elements (89.29%). The mean neighborhood support/cohesion/social capital was 9.69 (SD = 2.127).

Of the total sample of children without ADHD diagnosis (Table 1), most of them engaged in physical activity for 1 to 3 days (40.60%), had non-immigrant parents (66.10%), aged 6–11 years (50.59%), were females (51.24%), non-Hispanic White (50.33%), born in the U.S. (94.81%), lived in household with adults with college or higher degree (49.72%), lived in a safe neighborhood (94.99%), or lived in neighborhood without any detracting elements (74.84%). The average neighborhood support/cohesion/social capital was 9.74 (SD = 2.082).

The higher proportions of physical activity level, sociodemographic groups, and neighborhood characteristics among children without ADHD diagnosis were also observed among children with ADHD diagnosis, except based on sex and age. Among children with ADHD (Table 1), majority of them engaged in physical activity for 1 to 3 days (41.18%), had non-immigrant parents (79.07%), aged 12–17 years (57.64%), were males (68.50%), non-Hispanic White (58.55%), born in the U.S. (97.08%), lived in household with adults with college or higher degree (45.35%), lived in a safe neighborhood (93.57%), or lived in neighborhood without any detracting elements (72.61%). The average neighborhood support/cohesion/social capital was 9.37 (SD = 2.438).

### Trends in the prevalence of ADHD and current anxiety/depression

The prevalence of ADHD diagnosis was higher in 2021 than in 2019 and 2016 (Fig. 1). Thus, in general, the prevalence increased between 2016 or 2019 and 2021 (Fig. 1). The prevalence of current anxiety/depression diagnosis was more than three times higher among children with ADHD across all the years compared to those without ADHD and the overall population of the children (Fig. 2). Overall, Table 1 shows that the prevalence of current anxiety/depression was higher in children with ADHD (37.34%) than those without ADHD (7.42%).

### Differences in the prevalence of current anxiety/depression

There were significant differences in the prevalence of current anxiety/depression diagnosis across ADHD diagnosis status, physical activity, parental nativity, age, sex, race/ethnicity, child’s nativity, educational level of adults in the children’s households, neighborhood safety, neighborhood support/cohesion, and neighborhood detracting elements (Table 1). The prevalence was higher in children with ADHD diagnosis (37.34%, 95% CI = 35.93%, 38.77%), engaged in physical activity for 0 days (20.36%, 95% CI = 19.00%, 21.79%), aged 12–17 years (14.20%, 95% CI = 13.71%, 14.71%), females (11.81%, 95% CI = 11.33%, 12.30%), born in the U.S. (11.17%, 95% CI = 10.84%, 11.50%), lived in unsafe neighborhood (18.59%, 95% CI = 16.28%, 21.14%), or lived in neighborhood without any detracting elements (12.66%, 95% CI = 11.87%, 13.50%). Children of non-Hispanic White background had higher prevalence (13.19%, 95% CI = 12.81%, 13.57%), but not higher than those of other/multi-racial, non-Hispanic background. The prevalence for children of immigrant parents (5.89%, 95% CI = 5.28%, 6.55%) and children who lived in households with adults with less than high school education (7.59%, 95% CI = 6.36%, 9.04%) compared to their peers was the lowest. The average score of neighborhood support/cohesion was 9.20 (SD = 2.620) among the children with anxiety/depression diagnosis.

The prevalence of anxiety/depression among children without ADHD significantly differed based on physical activity, parental nativity, age, sex, race/ethnicity, child’s nativity, educational level of adults in the children’s households, neighborhood safety, neighborhood support/cohesion, and neighborhood detracting elements (Table 1). The highest prevalence of anxiety/depression was noted among children who had 0 days of physical activity (14.20%), non-immigrant parents (8.71%), aged 12–17 years (10.15%), females (9.16%), non-Hispanic White individuals (9.10%), born in the U.S. (7.58%), lived in households with adults with some college/technical education (7.92%) or *≥* college degree (7.89%), lived in unsafe neighborhood (13.26%), or lived in neighborhood without any detracting elements (8.64%). They had lower mean scores of neighborhood support/cohesion (9.27 [SD = 2.595]) compared to those without current anxiety/depression (9.77 [2.036]).

Among children with ADHD, there were statistically significant differences in the prevalence of current anxiety/depression based on only physical activity, age, sex, race/ethnicity, neighborhood safety, neighborhood support/cohesion, and neighborhood detracting elements (Table 1). Children who did not engage in physical activity (51.83%), aged 12–17 years (40.08%), females (43.93%), other/multi-racial non-Hispanic individuals (44.26%), lived in unsafe neighborhood (49.53%), or lived in neighborhood without any detracting elements (40.21%) had higher prevalence of current anxiety/depression diagnosis. Those who had current anxiety/depression had lower mean scores of neighborhood support/cohesion (9.10 [SD = 2.652]) compared to those without current anxiety/depression (9.53 [SD = 2.295]).

### Association between physical activity, parental nativity, and anxiety/depression

There were statistically significant associations between current anxiety/depression and ADHD diagnosis status, physical activity, and parental nativity, adjusting for the sociodemographic and neighborhood factors (Model 2, Table 2). Children with ADHD had higher odds of current anxiety/depression (AOR = 7.23, 95% CI = 6.67, 7.84) compared to those without ADHD. Engaging in physical activity for 1 to 3 days (AOR = 0.59, 95% CI = 0.53, 0.66), 4 to 6 days (AOR = 0.42, 95% CI = 0.37, 0.47), and daily (AOR = 0.41, 95% CI = 0.36, 0.47) was associated with lower odds of current anxiety/depression. The pairwise or multiple comparison tests showed that there were significant differences in anxiety/depression between almost all levels of physical activity (all *p* < 0.001), except the difference between 4 and 6 days vs. daily (p *≥* 0.05). Those with immigrant parents had lower odds of current anxiety/depression (AOR = 0.58, 95% CI = 0.51, 0.67) compared to those with non-immigrant parents. The multiple comparison tests revealed no significant difference between parents with unknown background vs. non-immigrant parents (p *≥* 0.05).

There were statistically significant interactions between ADHD diagnosis status and physical activity (Model 3, Table 2: *p* < 0.001) and ADHD diagnosis status and parental nativity (Model 4, Table 2: *p* = 0.038), adjusting for the covariates. However, the interactions between parental nativity and physical activity (Model 5, Table 2: *p* = 0.387) and the three-way interaction between ADHD diagnosis status, parental nativity, and physical activity (Model 6, Table 2: *p* = 0.763) were not statistically significant. That is, ADHD moderated the association between physical activity and current anxiety/depression; as shown in Fig. 3, the highest anxiety/depression probability was observed in children not engaging in physical activity, especially in children with ADHD diagnosis, but the lowest probability was noted for those engaging in daily physical activity among children without ADHD diagnosis. The computed multiple comparison tests showed significant differences in anxiety/depression diagnosis across some physical activity levels based on ADHD diagnosis status (all *p* < 0.001): there were significant differences between 0 days vs. all other days (1 to 3 days, 4 to 6 days, and daily), but not significantly different between 1 and 3 days vs. 4 to 6 days (p *≥* 0.05), 1 to 3 days vs. daily (p *≥* 0.05), or 4 to 6 days vs. daily (p *≥* 0.05) physical activity among children with ADHD diagnosis. Among children without ADHD diagnosis, the differences were statistically significant between 0 days vs. all other days (1 to 3 days, 4 to 6 days, and daily), 1 to 3 days vs. 4 to 6 days, and 1 to 3 days vs. daily but not significantly different between 4 and 6 days vs. daily physical activity (p *≥* 0.05). That is, anxiety/depression diagnosis declined between 0 days and all other days of physical activity (1 to 3 days, 4 to 6 days, or daily) for children with ADHD diagnosis, but decreased across almost all days of physical activity (except between 4 and 6 days vs. daily) among children without ADHD diagnosis.

ADHD also moderated the association between parental nativity and current anxiety/depression; as shown in Fig. 4, children whose parents’ background was unknown had the highest anxiety/depression likelihood, particularly children with ADHD, while children with immigrant parents had the lowest probability of anxiety/depression. Generally, anxiety/depression probability was highest for those whose parents’ background was unknown, followed by those with non-immigrant parents and immigrant parents among children with or without ADHD. The multiple comparison tests revealed significant differences in anxiety/depression across parental nativity groups by ADHD diagnosis status (all *p* < 0.001), except between non-immigrant parents vs. immigrant parents (p *≥* 0.05), parents with unknown background vs. immigrant parents (p *≥* 0.05), or parents with unknown background vs. non-immigrant parents (p *≥* 0.05) among children with ADHD, and parents with unknown background vs. non-immigrant parents (p *≥* 0.05) among children without ADHD.

Table 3 displays the relationship between physical activity, parental nativity, and current anxiety/depression based on ADHD diagnosis status, while accounting for sociodemographic and neighborhood factors. Among children without ADHD (Model 1, Table 3), engaging in physical activity for 1 to 3 days (AOR = 0.58, 95% CI = 0.51, 0.66), 4 to 6 days (AOR = 0.38, 95% CI = 0.33, 0.43), and daily (AOR = 0.36, 95% CI = 0.31, 0.42) was associated with lower anxiety/depression odds. Using multiple comparison tests, we observed significant differences in anxiety/depression across physical activity levels (all *p* < 0.001), except for the difference between 4 and 6 days vs. daily (p *≥* 0.05) not significant. Additionally, children with immigrant parents had lower odds of anxiety/depression (AOR = 0.53, 95% CI = 0.45, 0.62) compared to those with non-immigrant parents. While children with unknown parental backgrounds, however, had higher odds of anxiety/depression relative to children with non-immigrant parents, this difference was not statistically significant. The pairwise comparison tests showed significant differences between the parental nativity groups (all *p* < 0.001), apart from the difference between parents with unknown background vs. non-immigrant parents (p *≥* 0.05). There was no statistically significant interaction between parental nativity and physical activity (*p* = 0.239) among the children without ADHD, adjusting for the covariates (Model 2, Table 3).

Similarly, for children with ADHD diagnosis (Model 3, Table 3), engaging in physical activity for 1 to 3 days (AOR = 0.61, 95% CI = 0.51, 0.74), 4 to 6 days (AOR = 0.50, 95% CI = 0.40, 0.61), and daily (AOR = 0.51, 95% CI = 0.41, 0.64) was associated with lower odds of anxiety/depression. The multiple comparison tests indicated that the physical activity levels were significantly different from each other (all *p* < 0.001), except the difference was not significant between 1 and 3 days vs. 4 to 6 days (*p* ≥ 0.05), 1 to 3 days vs. daily (p *≥* 0.05), or 4 to 6 days vs. daily (p *≥* 0.05) among children with ADHD. Parental nativity was not statistically associated with anxiety/depression. The interaction between parental nativity and physical activity was not statistically significant (*p* = 0.896) among the children with ADHD, adjusting for the covariates (Model 4, Table 3).

## Discussion

This study examined the complex interactions among ADHD diagnosis status, anxiety/depression, physical activity, and parental background, adjusting for various sociodemographic and environmental factors, among children aged 6–17 years. Our results reveal noteworthy links between physical activity, parental background, and anxiety/depression in both children with and without ADHD, uncovering new insights and mental health disparities. Children with ADHD had about 7.2 times the odds of being diagnosed with anxiety/depression compared to their counterparts without ADHD, emphasizing the need to consider ADHD diagnosis status in treatment of anxiety/depression in children. Engaging in physical activity for at least one day was associated with 41–59% lower odds of anxiety/depression in the general children population. Among children with ADHD the odds were 39–50% lower, while among children without ADHD, the odds were 42–64% lower, compared to those not engaging in physical activity. These findings further highlight the potential benefits of engaging in at least some physical activity among children, especially those with ADHD. However, given that our study is cross-sectional, it is possible that children with no or less anxiety/depression engaged in more physical activity. These plausible bidirectional associations should be examined. Children with or without ADHD whose parents were immigrants were less often diagnosed with anxiety/depression than their native-born counterparts, supporting and contributing to the possible healthy immigrant effect hypothesis or the immigrant health paradox literature. This hypothesis suggests that immigrants have health advantages despite facing socioeconomic, immigration stressors, and other disadvantages [40, 60]. The lower odds of anxiety/depression diagnosis in immigrant children might be attributed to underdiagnosis of health outcomes (e.g., mental health outcomes) among immigrants, largely due to lack of access to culturally sensitive healthcare services [61]. Immigrants often face structural barriers (e.g., transportation, lack of insurance, high cost, language barriers, and discrimination) to accessing health services, which could affect their access to diagnosis [61]. Underdiagnosis may partly explain the immigrant health advantage [62], and thus need further examination. The results stress the importance of disaggregating data to address specific mental health disparities in subgroups, such as immigrants, for targeted interventions.

Notably, there was an increase in ADHD prevalence from 2016 or 2019 to 2021. Similar patterns in ADHD prevalence have been observed among children aged 4–17 years in other studies [53]. However, our prevalence estimates were higher than those in previous studies probably due to the inclusion of older children (6–17 years) in our study, as ADHD rates tend to be higher among older children [53, 54, 63]. The higher rates in older children in our study could also be due to the inclusion of ever diagnosis of ADHD and therefore likely to include children who no longer have ADHD diagnosis. In general, older children have a higher chance of having ever been diagnosed with ADHD partly because symptoms (e.g., difficulties with attention and organization) are more noticeable with increase in age and expectations [53, 54, 63–65]. The increase in ADHD prevalence aligns with a concurrent uptick in anxiety/depression, especially among children with ADHD, in line with previous studies that also noted elevated depression and anxiety in this group [66, 67]. These findings may also be explained by the compounded stressors and disruptions children experienced during these years, possibly due to global events like the COVID-19 pandemic [68].

Previous randomized trials and observational cohort studies revealed the protective effect of physical activity against anxiety/depression in children with and without ADHD [69, 70]. Similarly, we found that engagement in physical activity for 1 to 3 days, 4 to 6 days, or daily correlates significantly with lower odds of anxiety/depression, emphasizing the pivotal role of regular physical activity in mental health. Further research is needed to evaluate the patterns and impacts of physical activity on the mental health of children diagnosed with ADHD, to inform tailored physical activity and mental health interventions.

Personalized physical activity plans can boost children’s participation with reduced fatigue, stress, and improved mental health, benefiting non-immigrant children and those with ADHD. Parental nativity plays a significant role, with immigrant children, particularly those without ADHD, having lower anxiety/depression odds, potentially due to unique family structures, cultural values, and support systems [38]. However, the significant interaction effect of parental nativity and ADHD diagnosis also highlights the disparities in mental health outcomes based on familial backgrounds. This current study thus augments the literature with these findings, while identifying areas for future inquiry. Differential diagnosis and underdiagnosis of ADHD and anxiety/depression should be examined, especially among immigrant children due to underdiagnosis of health outcomes (e.g., mental health) in immigrant populations [61, 62, 75]. It is possible that some immigrant children had anxiety or depression that were not diagnosed by a healthcare provider.

Diverging patterns in anxiety/depression disorder based on sociodemographic and neighborhood factors unveil the multi-layered complexity underlying mental health disparities. For instance, females, especially those without ADHD, were more susceptible to anxiety/depression disorder than their male counterparts, pointing to potential gender-based vulnerabilities [48, 76]. This study also shows that racial/ethnic minority children (non-Hispanic Black, Asian, and Hispanic children) had lower odds of anxiety/depression disorder compared to their non-Hispanic White peers. These findings contrast other studies that found a higher prevalence of depression and anxiety disorders among pediatric minoritized communities [77, 78]. Diverse factors such as the intricate interplay of racial, cultural, and socioeconomic factors may impact the variances observed. It is, therefore, essential to consider these intersections in children’s mental health research and effective treatment or management strategies to reduce mental health disparities and burdens. It should be noted that our study only addressed self-reported diagnosed anxiety/depression disorder and therefore may be affected by bias regarding access to diagnosis, possibly resulting in underdiagnosis in some subgroups particularly minority populations [77].

Furthermore, we found that higher educational attainment of adults in a child’s household correlated with higher odds of anxiety/depression in children, notably those with ADHD diagnosis. This contradicts prevailing assumptions and findings that associate higher education with better mental health outcomes, prompting a reconsideration of conventional wisdom regarding educational attainment and mental well-being [79, 80]. This finding may be explained by diverse factors, including parental pressure and expectations among children living with adults with college or higher education and high school certificate or GED among individuals with and without ADHD, respectively [81]. However, differential access to mental health diagnosis services may have also influenced anxiety/depression diagnosis [61, 62, 75, 82], and therefore not the direct result of educational attainment of adults in a child’s household. Nonetheless, educational attainment is one of the socioeconomic determinants of access to quality opportunities and mental health services for children [77, 83–86].

The study’s cross-sectional design limits our ability to establish causation among ADHD diagnosis status, anxiety/depression, physical activity, parental nativity, and other factors. The observed associations do not imply causality. Self or parent-reported data may introduce recall and social desirability biases, leading to under and overestimation of the exposures and outcomes impacting interpretations. For instance, because anxiety and depression were measured by asking parents if a doctor or health professional told them their child had anxiety or depression disorder, there may be underreporting or overreporting of the child’s diagnosis due to recall challenges or social desirability bias. These biases could have resulted in the overestimation of the immigrant health paradox effect. Nonetheless, a similar health advantage or paradox has been observed among U.S. adults [87]. ADHD is a challenging condition to diagnose and therefore there is a possibility of its differential diagnosis [75], which may affect our observed associations. Heterogeneity in ADHD diagnosis and treatment among children may also affect outcomes. Underdiagnosis of health outcomes among immigrants, which may be due to lack of access to healthcare services [61], might have contributed to the lower odds of anxiety/depression diagnosis in immigrant children. Variations in diagnostic criteria, treatments, and medication adherence were not accounted for. Categorizing parental nativity may not fully encompass diverse immigrant experiences, potentially influencing associations with children’s mental health outcomes.

## Conclusions

This study revealed a complex interplay between ADHD diagnosis status, physical activity, and parental nativity. Our study also builds on the literature on the association between mental health and physical activity or parental nativity by exploring ADHD diagnosis status as a moderator. The findings indicate that significant mental health benefits can be realized among children with and without ADHD, particularly those with ADHD and non-immigrant parents, by engaging in regular physical activity. This underscores the potential protective effect of physical activity on children’s mental health, aligning with a growing body of evidence advocating its incorporation into comprehensive mental health strategies. This study also contributes to the evolving immigrant health paradox literature on children.

## Data Availability

All data generated and/or analyzed in this study are de-identified and publicly available at https://www.census.gov/programs-surveys/nsch/data/datasets.html.

## Abbreviations

PA: Physical activity
ADHD: Attention-Deficit/Hyperactivity Disorder
NSCH: National Survey of Children’s Health
HRSA MCHB: Health Resources and Services Administration’s Maternal and Child Health Bureau
STROBE: Strengthening the Reporting of Observational Studies in Epidemiology
